# Optimizing the Growth Conditions of the Selected Plant-Growth-Promoting Rhizobacteria *Paenibacillus* sp. MVY-024 for Industrial Scale Production

**DOI:** 10.3390/biology11050745

**Published:** 2022-05-13

**Authors:** Justina Kaziūnienė, Raimonda Mažylytė, Aurimas Krasauskas, Monika Toleikienė, Audrius Gegeckas

**Affiliations:** 1 Institute of Agriculture, Lithuanian Research Centre for Agriculture and Forestry, LT-58344 Akademija, Lithuania; monika.toleikiene@lammc.lt; 2 Life Sciences Center, Institute of Biosciences, Vilnius University, LT-10257 Vilnius, Lithuania; raimonda.mazylyte@gmail.com (R.M.); audrius.gegeckas@gf.vu.lt (A.G.); 3 St. Ignatius Loyola College, LT-44286 Kaunas, Lithuania; aurimas.krasauskas@ilk.lt

**Keywords:** diazotrophs, nitrogen, *Paenibacillus* spp., biomass, fermentation, optimization

## Abstract

**Simple Summary:**

Nitrogen is one of the most important elements for plant growth and development. However, irrational fertilization causes many environmental problems: high rates of nitrogen fertilizers change the soil pH, encourage nitrate and nitrite accumulation in plants and the soil, leached nitrogen compounds cause water eutrophication and drinking water contamination, and gaseous losses of nitrogen contribute to global warming. The biological nitrogen fixation (BNF) process, in which atmospheric nitrogen is converted to ammonia by microorganisms, has a significant role in the global nitrogen cycle and agriculture. Nitrogen-fixing-bacteria inoculants could help to reduce the losses of consistently rising prices of mineral fertilizers and help to implement green revolution strategies. In this research, we found the bacteria strain *Paenibacillus* sp. MVY-024 that has a positive impact on nitrogen accumulation in spring wheat and was easily applied on an industrial scale.

**Abstract:**

In this study, thirteen isolates, which were possibly expected to fix nitrogen, were isolated from soil and pea root nodules and identified by the gene analysis of 16S rDNA sequences. Two of these isolates that were able to form endospores and grow on nitrogen-free media were selected for spring wheat development research. The isolate *Paenibacillus* sp. S7 identified as *Paenibacillus polymyxa* was found to significantly increase the amount of ammonium and mineral N amounts in the soil. Furthermore, increased nitrogen accumulation in grains and a chlorophyll index were obtained after wheat treatment. *Paenibacillus* sp. S7 isolate was selected for further studies and the accession number MT900581 and strain name MVY-024 in NCBI nucleotide bank for this isolate were assigned. During the cultivation of *Paenibacillus* sp. MVY-024, sugarcane molasses and a yeast extract were determined as the most suitable carbon and nitrogen sources, whose optimal concentrations were 100 g L^−1^ and 10 g L^−1^, respectively. The optimal pH range for the cell culture was between 6.5 and 7.0, and the optimal air flow rate was 0.4 vvm. It was found that the air flow has an effect on biomass production and endospore formation. After *Paenibacillus* sp. MVY-024 biomass cultivation optimization, the cultured cell number was, on average, 2.2 × 10^9^ cfu m L^−1^.

## 1. Introduction

One of the main purposes of modern agriculture is to reduce the use of mineral fertilizers. Because of this reason, new species of bacteria that can fix nitrogen from the atmosphere are constantly being sought. Most industrial products for nitrogen fixation contain non-sporulating strains, which are sensitive to harmful environmental factors and difficult to survive. For superior microbial fertilizers, it is important to select nitrogen-fixing bacteria strains that are effective, competitive, and resistant to environmental factors. The purpose of this investigation is to find novel nitrogen-fixing strains that form endospores and promote plant growth. *Paenibacillus polymyxa* is Gram-positive endospore-forming, plant-growth-promoting, and biological-nitrogen-fixing rhizobacteria that have a high potential as a bacterial fertilizer in agriculture [1,2]. These bacteria are found in the rhizosphere and also as the root and stem endophytes of various crop plants, especially wheat [3,4]. Nitrogen fixation, the production of auxin and other indolic and phenolic compounds, siderophores, and phosphate solubilization directly promote plant growth and development [5,6,7]. Biofilm formation on plant roots and the production of antibiotics by *P. polymyxa*, such fusaricidins, polymyxins, and many others [8,9,10], ensure a strong effect against a variety of pathogenic microorganisms and also indirectly promote plant growth [11,12]. The *P. polymyxa* microbial product is easily compatible with mineral fertilizers and chemical plant protection products, as *P. polymyxa* cells survive in the environment for a long period of time because of spore formation under adverse conditions [13].

There is a lot of scientific research about the optimization of the production of secondary metabolites during *P. polymyxa* fermentation; however, the biomass cultivation of these bacteria is poorly described and a complicated process. Endospore formation is an important factor that ensures microbial product stability and efficiency on the soil and plants [14]. The main problem in the biomass cultivation process of *P. polymyxa* is that, after the exponential fermentation phase, many cells are not able to form endospores and die. Fermentation conditions and media optimization help to improve endospore formation, increase viable cell numbers in the final microbial product, provide an opportunity to ensure production efficiency, and reduce the product price, making the product more attractive for smallholder farmers [15,16]. 

In this study, a novel endospore-forming strain *Paenibacillus* sp. MVY-024 is isolated and identified as 99.71% homologous to *P. polymyxa*. Significantly increased ammonium and mineral N amounts in the soil and higher protein accumulation in grains after *Paenibacillus* sp. MVY-024 application proved that this strain is capable to fixing nitrogen from the atmosphere and has a potential as a nitrogen fixation product.

## 2. Materials and Methods

### 2.1. Sample Collection

Soil samples and pea seedlings were collected in the Panevezys region, Lithuania [55°42′06.4″ N 24°16′04.7″ E]. Soil samples were collected from fields where wheat grows; 10 soil samples were collected randomly with a 0–30 cm depth of the soil. All samples were mixed. The pea seedlings were uprooted from the pea field; 20 seedlings were collected randomly from different field locations. The isolation of microorganisms was performed on the same day when the samples were collected. The experimental research on plants, including the collection of plant material, complied with the relevant institutional, national, and international guidelines and legislation. The appropriate permissions for collection of plants were obtained for the study.

### 2.2. Isolation of N_2_-Fixer Rhizobacteria

During the isolation of the diazotrophic soil microorganisms, 1 g of the soil sample was added to 10 mL of sterile deionized water and suspended. The soil suspension was incubated for 20 min at 30 °C temperature, at 130 rpm in a shaking incubator. After incubation, the suspension was diluted in 10^−3^, 10^−4^, 10^−5^, and 10^−6^ series according to the serial dilution method and 100 µL of each dilution was plated onto solid NF media using the spread plate method. Plates were incubated in a bacteriological incubator at 30 °C for 48 h [17]. Each isolate was tested for growth in Ashby’s Mannitol [18], Winogradsky’s [19], NF [20], and NFB [21] nitrogen-free agar media.

During the isolation of diazotrophic microorganisms from pea root nodules, the roots were washed under running tap water for 10 min, then using tweezers, pink color nodules were carefully taken from the roots. The nodule surface was disinfected for 30 s in 70% ethanol solution and washed 5 times with sterile deionized water. Then, the nodules were treated for 1 min in a 3% sodium hypochlorite solution and washed 10 times with sterile deionized water [22]. The disinfected nodules were transferred into a tube with 5 mL sterile deionized water and homogenized using a sterile glass rod. The prepared bacterial suspension was diluted in 10^−3^, 10^−4^, 10^−5^, and 10^−6^ series according to the serial dilution method and 100 µL of each dilution was plated onto solid NF media using the spread plate method. Plates were incubated in a bacteriological incubator at 30 °C for 48 h. Each colony was purified by streaking on solid NF media.

### 2.3. Nitrogen Fixation Capabilities with N-Free Media

All thirteen isolated strains were tested for the growth by streak plate method on different Ashby’s Mannitol, Winogradsky’s, and NF nitrogen-free agar media. The growth of bacterial biomass was assessed visually. NFB semi solid media was used for the nitrogen fixation test. A single colony of the isolate was inoculated in a vial containing a semi-solid NFB medium and incubated at 30 °C for five days. The positive nitrogen fixation is observed if the medium color changes from green to blue and a pellicle is formed. Color changes are caused by ammonia production during nitrogen fixation. Ammonia is a weak base that changes the medium pH. The alkalization of the culture medium is observed by using a bromothymol blue indicator [21].

### 2.4. Endospore Formation Test

The ability of bacteria to form endospores was evaluated microscopically and by the standard plating procedure. Suspensions of all strains were incubated in a laboratory water bath at 75 °C for 15 min and 1 mL of the heat-treated bacterial suspension was plated onto a MPA agar medium and incubated in a bacteriological incubator for 48 h at 30 °C. Afterwards, incubation bacterial colonies were counted and phenotype traits were tested [23].

### 2.5. Molecular Identification of Isolated Microorganisms

Isolate identification was carried out using partial 16S rDNA sequence analysis. All partial 16S rDNA sequences were determined by PCR with primers 8 F (5′-AGAGTTTGATCCTGGCTCAG-3′) and 1492R (5′-GGTTACCTTGTTACGACTT-3′) [24] and compared to the EzBiocloud identification service. To determine the phylogenetic relationships, a phylogenetic tree was constructed by comparing obtained 16S rDNA sequences with related type strains 16S rDNA sequences. A phylogenetic tree was constructed using MEGA 5.0 software. According to the results of soil nitrogen changes and spring wheat experiments, the accession number MT900581 and strain name MVY-024 for *Paenibacillus* sp. S7 in NCBI nucleotide bank were assigned.

### 2.6. Vegetative Growth Promotion in Wheat Plants by Paenibacillus spp.

The vegetative pot experiment was conducted in 2019–2020, under regulated greenhouse conditions. Soil was used from an organically managed field 0–20 cm from the top. The soil was a loamy Endocalcaric Epigleyic Cambisol (Drainic, Loamic) CM-can.glp-dr.lo [25] and was characterized as having a high fertility level with 3.9% humus, 151 mg N kg^−1^, 93 mg P kg^−1^, and 156 mg K kg^−1^. The soil (5 kg dry weight pot^−1^) was filled in 8.5 l PVC pots. Two microbiological products of *Paenibacillus* sp. S1 and *Paenibacillus* sp. S7 were spread on the topsoil by watering in the form of a water solution containing 400 mL H_2_O and a 38 µL suspension of bacteria. There were 1.5 × 10^8^ cfu per pot. The solution was applied two times: at the beginning and 3 weeks later. Only water was applied on the control treatment. The spring wheat “Collada” was sown in the pots, in five replications, with 10 plants per each pot. The growing conditions in the greenhouse during the experiment were controlled: 16/8 h light/dark; photosynthetically active radiation at canopy level of 600 mol m^2^ s^−1^; temperature 20 °C day and 16 °C night time; irrigation of 200–400 mL per pot, 2 times a week.

### 2.7. Soil and Plant Analysis

The ammonium (N-NH4) in the soil was measured spectrophotometrically (with Cary 60 UV-Vis, USA) 4 days, 2 weeks and 2 months after the first *Paenibacillus* spp. application. The mineral nitrogen (Nmin) content was measured after 2 months as the sum of N–NO3 and NH4. Spring wheat in the pots was analyzed during BBCH 37 (SPAD) and BBCH 87 growing stages for the grain yield per plant, thousand kernels weight (TKW), proteins in grain and kernels per spike. The concentration of protein and N yield in grain was measured using the Kjeldahl method [26].

### 2.8. Optimization of Carbon and Nitrogen Sources for Paenibacillus sp. MVY-024 Cultivation

The optimization of carbon and nitrogen sources was performed in a shake-flask experiment using an AF medium (10 g yeast extract, 40 g fructose, 0.2 g MgSO_4_·7H_2_O, 0.4 g NaCl, 0.5 g K_2_HPO_4_, 0.1 g FeSO_4_·7H_2_O, in 1000 mL deionized water, pH 6.6–7.0), which was also used for inoculum preparation and control. Glucose, sucrose, glycerol, mannitol, molasses, and starch were selected as carbon sources and soybean, peptone, urea, ammonium sulphate, casein peptone, agropeptone, yeast extract, and meat extract were used as a nitrogen source (Table 1). Considering that 100 g of cane molasses contains around 70 g of sugars, the molasses concentration was recalculated. During the optimization of the carbon source, the selected nitrogen source was the yeast extract.

Under sterile conditions, 200 mL of the sterile medium was poured to a 1 L Erlenmeyer flask and 2 mL of fresh *Paenibacillus* sp. MVY-024 inoculum was inoculated in the flask. The concentration of *Paenibacillus* sp. MVY-024 cells in the initial inoculum was 5.0 × 10^6^ cfu m L^−1^. All samples using different carbon or nitrogen sources were incubated for 24 h at 30 °C, at 130 rpm in a shaking incubator. After incubation, the number of bacterial cells in the suspensions was determined on solid MPA (20 g meat extract, 5 g glucose, 10 g agropeptone, 20 g agar in 1000 mL deionized water, pH 6.6–7.0) medium according to the serial dilution–spread plate method. The plates were incubated in a bacteriological incubator at 30 °C for 48 h.

### 2.9. pH and Temperature Optimization

To determine the optimum initial pH for *Paenibacillus* sp. MVY-024 biomass production, the pH of the medium was adjusted to the desired pH by adding 1 M HCl and 1 M NaOH before sterilization. The pH values of the AF medium were 6.0, 6.5, 7.0, 7.5, 8.0. a total of 200 mL of sterile medium was poured to a 1 L Erlenmeyer flask and 2 mL of fresh *Paenibacillus* sp. MVY-024 inoculum was inoculated. All samples using different pH values were incubated for 24 h at 30 °C, at 130 rpm in a shaking incubator. After incubation, the number of bacteria cells in suspensions was obtained on a solid MPA medium according to the serial dilution–spread plate method. The plates were incubated in a bacteriological incubator at 30 °C for 48 h.

After the determination of the optimal pH for biomass production, the same shake-flask experiment was repeated in the same conditions, but using different temperature values: 28 °C, 30 °C, 32 °C, 34 °C, and 36 °C. The samples were diluted in serial dilutions, plated on a MPA solid medium, and incubated in bacteriological incubator at 30 °C for 48 h. The number of bacterial colonies was counted.

### 2.10. Optimization of Air Flow

*Paenibacillus* sp. MVY-024 air flow optimization was performed in a fermenter (EDF 5.4_1). Cell culturing was performed in an optimized AF medium, using molasses and yeast extract as carbon and nitrogen sources in determined optimal concentrations, respectively. Based on the results of the temperature and pH optimization, biomass culturing was performed at 32 °C when the pH value was 7.0 ± 0.5. During the fermentation process, the pH value of the medium was adjusted by using automatic titration with 2 M NaOH and 2 M H_2_SO_4_ and antifoam was used to reduce foaming. Feeding was started after 8 h of fermentation and was fed into the bioreactor for 5 h. Every hour, 60 mL of feeding was used. During air flow optimization, the same partial pressure of oxygen and different air flow rates were selected. The partial pressure of oxygen in the medium was 20 ± 2 with the air flow rates of 0.1 vvm, 0.2 vvm, 0.4 vvm, 0.8 vvm, 1.2 vvm, 1.6 vvm, and 2.0 vvm. The air was supplied to the bioreactor through a 0.2 µm pore size filter. The stirrer was set to automatic mode, from 45 to 800 rpm. A total of 200 mL of inoculum and 3 L of optimized AF medium were used for fermentation. During the process, parameters such as temperature, pH, agitation rate and partial pressure of oxygen were monitored and recorded. The optimal cell culturing time in the bioreactors was about 70 h; during this time, all fermentable bacterial cells had to form endospores.

### 2.11. Statistical Analysis

All statistical analyses were performed using SAS software version 9.4 (SAS Institute Inc., Copyright © 2002–2010). The graphical representation of the data was performed using the GraphPad software package. Homogeneity and normality were verified using Bartlett’s test. Experimental data were analyzed by a one-way analysis of variance (ANOVA) and mean comparisons between treatments were performed using Duncan’s mean separation test. The smallest significant difference R05 was calculated using a probability level of *p* < 0.05.

## 3. Results

### 3.1. Isolation and Description of the Physiological Properties of the Isolates

Thirteen isolates were isolated from soil and pea root nodules on NF agar media. Each isolate was tested for growth in Ashby’s, Winogradsky’s, and NF nitrogen-free agar media (Table 2).

Three isolates, S1, S3, and S7, showed quite intensive growth in all media. Isolates S3 and S7 demonstrated the most intensive growth on the NF agar media and at an average on the Ashby’s and Winogradsky’s nitrogen free media (Table 2). Isolate S1’s growth in the Ashby’s medium was better compared to that of S3 and S7, but growth in Winogradsky’s media was weaker. The isolates S4, S6, and R2 showed the weakest growth in all semi-selective media. NFB media showed that only half of the isolated bacteria formed a pellicle and possibly are capable of fixing nitrogen from the atmosphere; a positive nitrogen fixation reaction was demonstrated in the culturing of isolates S1, S2, S3, R3, and R4. The growth of isolates in each media was different because of the individual demand for salt and carbon sources.

Gram staining and the endospore determination method revealed that nine isolates are Gram positive and are capable of forming endospores, with the other four isolates being Gram negative and not being able to produce endospores (Table 2). Given the results of isolate growth on semi-selective media, nitrogen fixation reaction and the fact that microorganisms that are capable of forming endospores are much more resistant to adverse environmental factors [27,28], the isolates S1 and S7 were selected for spring wheat growth promotion investigation.

### 3.2. Phylogenetic Analysis

The phylogenetic analysis based on 16S rDNA sequences showed that the S4, S5, S6, S8, S9, R1, and R2 isolates belong to *Bacillus* spp.; the S2 and S3 isolates are members of *Ensifer* spp.; the R3 isolate belongs to *Lelliottia* spp.; R4 is a member of *Rhizobium* spp.; and the target S1 and S7 isolates belong to *Paenibacillus* spp. (Table 3).

**Figure 1:**
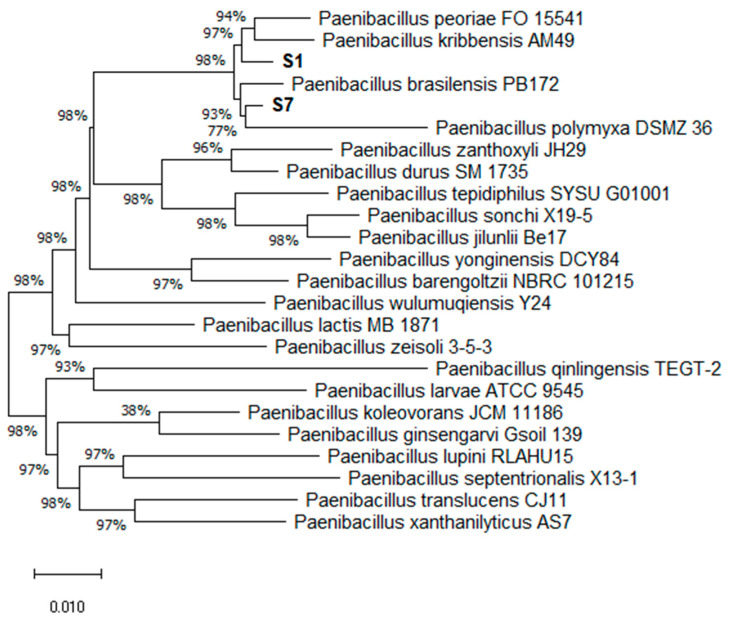
The phylogenetic relationships of *Paenibacillus* sp. S1 and *Paenibacillus* sp. S7 within the genus *Paenibacillus* spp. investigated using 16S rRNA gene sequence analysis. The phylogenetic tree was constructed using MEGA 5.0 software package, by the neighbor-joining statistical method with 1000 replicates of bootstrap. The scale bar illustrates 0.01 substitutions per nucleotide position.

The isolates’ 16S rRNA sequences were compared with the 16S rRNA gene sequences of related type strains. Based on the identification results of the obtained 16S rRNA gene sequences, a phylogenetic tree was constructed (Figure 1). The phylogenetic tree showed that both isolates belong to different *Paenibacillus* species. The isolate S1 is closely related to the typical *P. peoriae* FO 15,541 and *P. kribbensis* PB172 strains. The isolate S7 strain is related to the typical *P. polymyxa* DSMZ 36 strain.

The comparison of the 16S rDNA sequence obtained from the S1 and S7 isolates showed that both isolates belong to different *Paenibacillus* species. The isolate S1 is closely related to *P. peoriae* FO 15541, with a percent identity of 99.28%, and to *P. kribbensis* PB172 with a high percent identity of 98.48%. The isolate S7 strain is homologous to the *P. polymyxa* DSMZ 36 strain, with a percent identity of 99.71%.

### 3.3. Soil Nitrogen Changes as Affected by Paenibacillus sp. S1 and Paenibacillus sp. S7

Four days after bacteria application, the amount of N-NH4 in the soil did not vary significantly among the treatments (Table 4). However, significantly higher amounts (*p* < 0.05) of N-NH4 were observed after 2 weeks for both *Paenibacillus* spp. strains compared with the control treatment. After a 2-month wheat growth period (BBCH 87), the amount of N-NH4 was lower, but *Paenibacillus* sp. S7 held the highest ammonium and mineral N amounts in the soil.

The nitrogen accumulation in grains was significantly higher (*p* < 0.05) for the treatment with *Paenibacillus* sp. S7, compared with *Paenibacillus* sp. S1, and was not significant, but was also higher compared with the control (Table 5). Chlorophyll, in the leaf tissues indicating N, was also significantly higher for the control and *Paenibacillus* sp. S7. All yield components, including grain yield per plant, thousand kernels weight (TKW), proteins in grain, and kernels per spike, differed significantly from the control, when the *Paenibacillus* spp. S7 strain was applied on the soil. After *Paenibacillus* sp. S7 application, grain yield was 9%, TKW was 5%, and protein content was 11% higher compared to the control. The effect of *Paenibacillus* sp. S1 was very similar to that of the control.

According to results of the soil nitrogen changes and spring wheat experiments, the accession number MT900581 and strain name MVY-024 for *Paenibacillus* sp. S7 in NCBI nucleotide bank were assigned. This strain *Paenibacillus* sp. MVY-024 was selected for biomass production.

### 3.4. Effect of Carbon and Nitrogen Sources on Paenibacillus sp. MVY-024 Biomass Production

**Figure 2:**
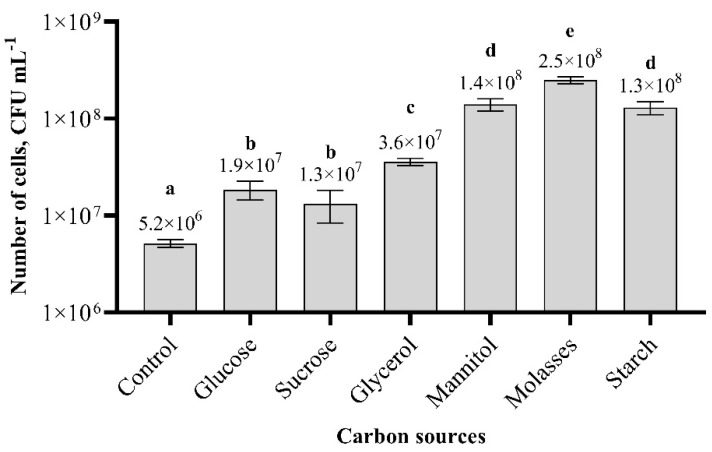
Effect of different carbon sources on *Paenibacillus* sp. MVY-024 biomass production. The values marked with the same letter have no significant difference at *p* ≤ 0.05.

The results obtained from the shake-flask experiments revealed that the different carbon and nitrogen sources have an effect on the amount of biomass production (Figure 2). The lowest biomass production was obtained using glucose, sucrose, and glycerol. Differences between glucose and sucrose variants were insignificant. The highest biomass production was in the nutrition medium with molasses, where the concentration of cells was 48 times higher compared to that of the control, in which the AF medium was used. The number of cells was 2.5 × 10^8^ cfu m L^−1^. Biomass production in media using mannitol and starch as carbon sources showed approximately a 2-fold lower number of cells compared to that achieved using molasses.

**Figure 3:**
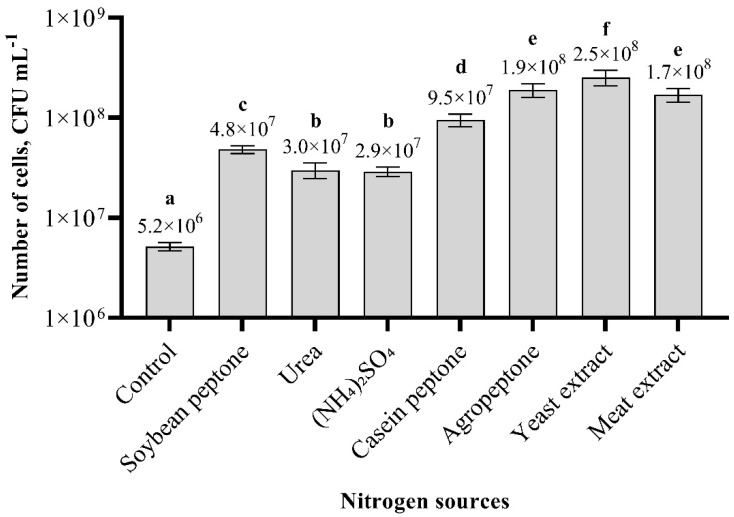
Effect of different nitrogen sources on *Paenibacillus* sp. MVY-024 biomass production. The values marked with the same letter have no significant difference at *p* ≤ 0.05.

The results obtained from the shake-flask experiments revealed that the weakest biomass growth was found in samples using urea and ammonium sulphate as nitrogen sources (Figure 3). The highest biomass production was achieved when the yeast extract was used. The number of cells using the yeast extract as a nitrogen source was 2.5 × 10^8^ cfu m L^−1^, which is 48 times higher than that of the control. Agropeptone and the meat extract showed a similar result, which is not significantly different. Based on the results obtained, the most suitable nitrogen source for *Paenibacillus* sp. MVY-024 biomass cultivation is the yeast extract.

**Figure 4:**
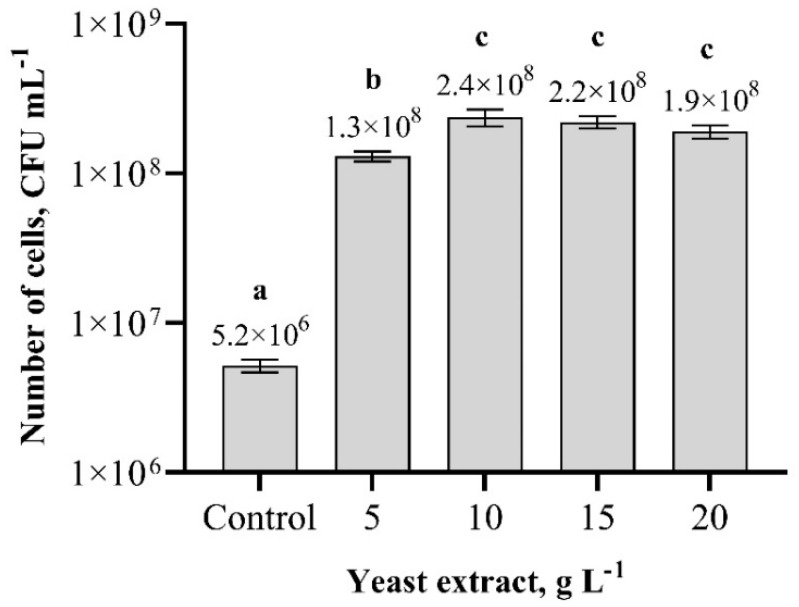
Effect of different concentrations of yeast extract on *Paenibacillus* sp. MVY-024 biomass production. The values marked with the same letter have no significant difference at *p* ≤ 0.05.

Studies have shown that the most suitable carbon and nitrogen sources for *Paenibacillus* sp. MVY-024 biomass production are sugar cane molasses and yeast extract, respectively, so these substrates were chosen for further studies. To determine the optimal concentrations of molasses and yeast extract for *Paenibacillus* sp. MVY-024 biomass cultivation, research was continued using the shake-flask experiments. The results indicate that the lowest cell biomass production was obtained when the concentration of yeast extract was 5 g L^−1^, and increasing the yeast extract concentration from 10 g L^−1^ to 20 g L^−1^ showed a higher biomass production, but differences between these variants were insignificant (Figure 4). However, the optimal yeast extract concentration for *Paenibacillus* sp. MVY-024 biomass production is 10 g L^−1^, and the number of cells on average in this concentration was 2.4 × 10^8^ cfu m L^−1^.

**Figure 5:**
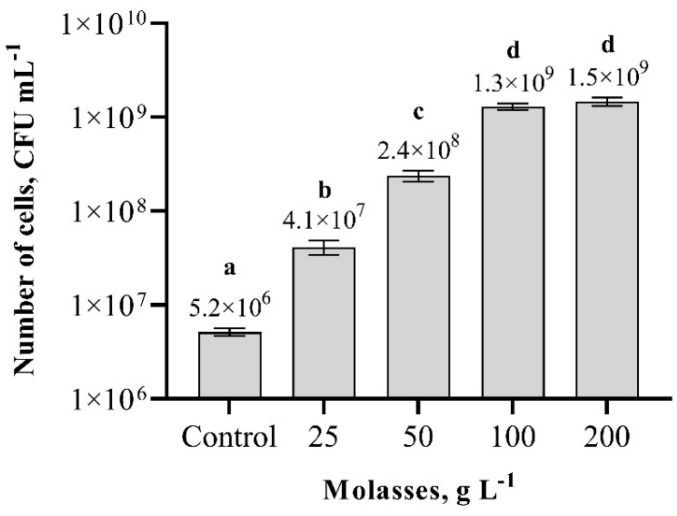
Effect of different concentrations of molasses on biomass production of *Paenibacillus* sp. MVY-024. The values marked with the same letter have no significant difference at *p* ≤ 0.05.

Experiments with different molasses concentrations showed that the lowest number of cells were when molasses concentration was 25 g L^−1^ (Figure 5). Nevertheless, while increasing the molasses concentration to 100 g L^−1^, the number of cells increased more than 250-fold compared to the control sample and the cultured bacterial biomass of the cultured cells was on average 1.3 × 10^9^ cfu m L^−1^. When the molasses concentration was increased to 200 g L^−1^, no significant difference was observed, and the number of cells obtained was the same as the number of cells in the sample, where molasses concentration was 100 g L^−1^. In conclusion, the optimal concentration of molasses for *Paenibacillus* sp. MVY-024 biomass cultivation is 100 g L^−1^.

### 3.5. Optimization of Nutrition Broth pH and Temperature for Paenibacillus sp. MVY-024 Biomass Production

**Figure 6:**
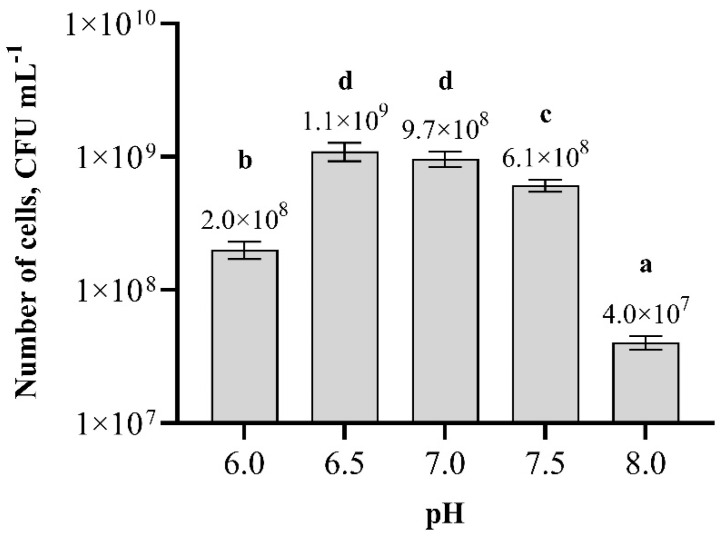
Effect of pH values on the biomass production of *Paenibacillus* sp. MVY-024. The values marked with the same letter have no significant difference at *p* ≤ 0.05.

In this experimental study, by adjusting the pH values of the nutrition broth, it was found that the most suitable pH values for *Paenibacillus* sp. MVY-024 biomass growth are 6.5–7.0 (Figure 6). The number of cells in these pH values was 1.1 × 10^9^ cfu m L^−1^ and 9.7 × 10^8^ cfu m L^−1^, and the difference was insignificant. At higher or lower pH values, *Paenibacillus* sp. MVY-024 biomass production was decreased.

**Figure 7:**
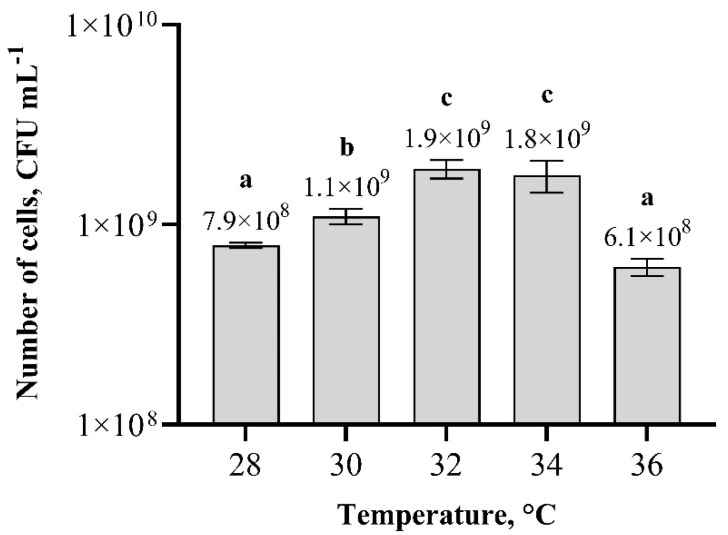
Effect of temperature on the biomass production of *Paenibacillus* sp. MVY-024. The values marked with the same letter have no significant difference at *p* ≤ 0.05.

Experiments using different temperatures for *Paenibacillus* sp. MVY-024 biomass production showed that the highest cell number was achieved when the temperature was around 32 °C and 34 °C (Figure 7). The number of cells at these temperatures was 1.9 × 10^9^ cfu m L^−1^ and 1.8 × 10^8^ cfu m L^−1^, respectively; the difference was insignificant. During biomass cultivation at higher or lower temperatures, the number of *Paenibacillus* sp. MVY-024 cells decreased. The difference in the number of cells, during biomass cultivation at 28 and 36 °C temperatures, was insignificant.

### 3.6. Optimization of Air Flow for Paenibacillus sp. MVY-024 Biomass Production

**Figure 8:**
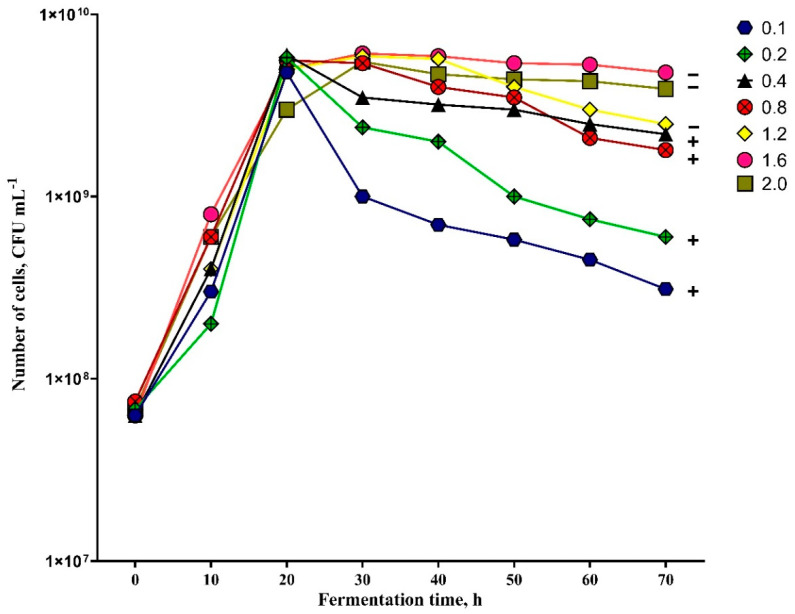
Effect of air flow on the biomass production of *Paenibacillus* sp. MVY-024. Differences between averages are significant at *p* ≤ 0.05.

The results of the experiment show that the airflow feed rate for biomass production and spore formation is significant (Figure 8). Biomass production was similar in all samples for the first 20 h of fermentation, where different air flow rates were used. In several fermentations, the number of bacteria cells reached a maximum value. According to the 30 h results, the number of cells started to decrease in the samples where air flow was 0.8 vvm and lower, and in the samples where air flow was 1.2 vvm and higher, the number of cells increased compared to the results of 20 h. The samples where air flow was 0.1 vvm, 0.2 vvm, 0.4 vvm, and 0.8 vvm at 30–40 h fermentation hours showed the first endospore-forming cells. All cells in fermentations, where air flow was 0.8 vvm and lower, formed free endospores until 70 h of fermentation. Cells did not form any endospores in the samples where air flow was 1.2 vvm and higher, until 70 h of fermentation; fermentations continued until 110 h, and around 60–80% of vegetative cells formed spores, but the number of cells decreased, and less than 1 × 10^8^ cfu m L^−1^ was obtained, so the fermentations were stopped.

The results show that the highest number of cells in the final sample was obtained when the air flow was 1.6 vvm and 2.0 vvm. The average number of cells was 4.8 × 10^9^ cfu m L^−1^ and 3.9 × 10^9^ cfu m L^−1^ in these fermentations, respectively. However, during fermentations where airflow rates were 1.6 vvm and 2.0 vvm, any endospore formation was not achieved in over 70 h, so it could be that these airflows are not suitable for culturing *Paenibacillus* sp. MVY-024 cells. The optimal air flow for the biomass production of *Paenibacillus* sp. MVY-024 is 0.4 vvm; during this fermentation process, all cells formed endospores in 70 h and the number of cells obtained was the highest compared to that of other successful fermentations. The number of spores was averaged at 2.2 × 10^9^ cfu m L^−1^ in the final sample.

## 4. Discussion

There are many studies that prove the positive effect of *P. polymyxa* on plants. Russian scientists demonstrated that *P. polymyxa* CCM 1465 and *P. polymyxa* 92 strains increase the mitotic index of the root cells by 1.2- and 1.6-fold after inoculation on wheat seedlings. It was determined that these two strains and their produced EPSs promoted wheat growth and development, increasing root and shoot length up to 22% and root and shoot dry weight up to 28% compared with the control [29]. A study with the *P. polymyxa* SbCT4 strain also confirmed a positive effect on wheat and maize growth promotion; root length and dry weight were significantly higher compared to the control [30]. Canola and tomato growth parameters were significantly better after *P. polymyxa* P2b-2R treatment. Tomato seedlings inoculated with P2b-2R strain were assimilated with nearly 90% more biomass than controls, nearly 40% longer than controls, and fixed nearly 17% of nitrogen from the atmosphere [31]. Our novel *Paenibacillus* sp. MVY-024 strain also showed a positive effect on soil nitrogen changes and spring wheat growth parameters. After two months of *Paenibacillus* sp. MVY-024 application on plants, ammonium was 2.5-fold and mineral N amounts 1.4-fold significantly higher compared with the those of the control. After *Paenibacillus* sp. MVY-024 application, chlorophyll was 2%, grain yield was 9%, TKW was 5%, and protein content was 11% higher compared to the control. The significant higher nitrogen accumulation in the soil and plants after the *Paenibacillus* sp. MVY-024 treatment proved that this strain could fix nitrogen from the atmosphere and promote plant growth.

During the optimization of carbon and nitrogen sources, it was determined that sugarcane molasses at 100 g L^−1^ and yeast extract at 10 g L^−1^ are the most suitable for *Paenibacillus* sp. MVY-024 biomass production, as the number of cells spores in the final product was 2.2 × 10^9^ cfu m L^−1^. In Gong and colleagues’ studies, where carbon and nitrogen sources were optimized for *P. polymyxa* BY-28 cell growth, the best nitrogen sources were determined to be polypeptone and bean powder, given the results of 3.1 × 10^8^ cfu m L^−1^ and 3.0 × 10^8^ cfu m L^−1^, respectively; the cell number with a yeast extract was 2.8 × 10^8^ cfu m L^−1^. The most suitable carbon sources were indicated to be maltose at 3.3 × 10^8^ cfu m L^−1^ and glucose at 3.2 × 10^8^ cfu m L^−1^ [32]. After fermentation medium optimization, a cheaper medium composition was obtained. Sugarcane molasses is a very cheap carbon source; one liter molasses cost around EUR 20 cents. After fructose replacement to sugarcane molasses, the growth medium price became cheaper by 46%. Sugarcane molasses is a natural organic carbon source, but its composition varies with cane quality and the type of refining. So, sugarcane molasses composition variation could lead to differences in bacteria biomass production results. According to the results of J. Liu and his colleagues, optimizing the medium of *P. polymyxa* EJS-3, the maximum dry cell mass was obtained when the pH value was 6.0 33. The results of V. Raza revealed that the most suitable pH for *P. polymyxa* SQR-21 cell growth was 6.5 [33]. Our experimental study proved that the most suitable pH values for *Paenibacillus polymyxa* sp. MVY-024 biomass growth are 6.5–7.0 and the temperature is 32 °C. In previous studies for the cell culturing of *P. polymyxa* BY-28, the optimal temperature was 30 °C, 32 °C for 2,3-Butanediol production, 30 °C for *P. polymyxa* ZJ-9, 35 °C for *P. polymyxa* DSM 365 [34], and 37 °C for the *P. polymyxa* PM 3605 strain [35]. Based on the results obtained and those reported in the literature, the optimal media pH for *P. Polymyxa* growth and metabolite production is from 6.0 to 7.0; meanwhile, the temperature is from 30 °C to 37 °C, so the culture temperature and pH tolerance are specific for each *P. polymyxa* strain.

The scientific literature states that insufficient nutrients or unfavorable fermentation conditions promote sporulation. In our investigation, it was determined that the sporulation time was longer and spores’ number was lower in the samples where air flow was 1.2 vvm and higher, and this contradicts the data provided in the literature. Although there is no research about the effects of different airflow rates on *P. polymyxa* biomass production, some scientific publications have suggested that intensive aeration during the fermentation process increases the synthesis of acetoin and acetate, which can inhibit *P. polymyxa* biomass growth and the synthesis of other secondary metabolites [36,37,38]. It is possible that that acetoin and acetate production in higher air flow rates affected endospore formation. In this research, it was observed that, when the air flow was 1.2 vvm or higher, the volume of condensate increased proportionally with increasing air flow rate. Thus, as the air flow rate increases, a part of the fermentation product evaporates. Therefore, it is very important to select the optimum air flow for the fermentation process. 

The difference between industrial scale reactors and laboratory-scale bioreactors is capacity. Industrial scale reactor capacity can be from tens to thousands of liters. Although the fermentation line usually consists of several different capacity bioreactors, the fermentation process starts in the lowest capacity reactor. When the exponential cell growth phase is achieved, the fermentation product is inoculated into a larger capacity bioreactor, and the process is repeated until the maximum capacity bioreactor is achieved. Our obtained results about *Paenibacillus* sp. MVY-024 fermentation growth medium and fermentation parameters are easily applied on an industrial scale. Based on our fermentation protocol, similar *Paenibacillus* sp. MVY-024 biomass production results could be achieved at an industrial level.

It is very important to verify the safety of the release of *Paenibacillus* sp. MVY-024 strain into an environment in further studies. According to the European Regulation (EU) 2019/1009 on Biostimulants, “EU fertilizing products should be placed on the market only if they are sufficiently effective and do not present a risk to human, animal or plant health, to safety or to the environment when properly stored and used for their intended purpose, or under conditions of use which can be reasonably foreseen, that is when such use could result from lawful and readily predictable human behaviour”. However, according to this regulation, only four different genera, *Azotobacter* spp., *Mycorrhizal* fungi, *Rhizobium* spp., and *Azospirillum* spp., can be used as biofertilizers without specific control. Unfortunately, these taxonomic criteria do not guarantee the safety of the strains approved to be registered as safe, because these genera could include both pathogen and harmless strains. No pathogen has been identified in *Rhizobium* spp., *Azospirillum* spp., *Azotobacter* spp., and *Mycorrhizal* fungi, but it does not guarantee that they do not exist. In further studies, our isolated strain should be analyzed according to the Environmental and Human Safety Index (EHSI). This index reflects the impact of the strain on human health and its effect on the environment and indicates microorganism pathogenicity score from 0 to 100; if a microorganism scores less than 50, it is considered that the microorganism is safe and could be released to the environment [39,40].

## 5. Conclusions

We were able to isolate the novel strain *Paenibacillus* sp. MVY-024 in this study. Phylogenetic analysis based on 16S rDNA sequences showed that this strain is closely related to the *Paenibacillus polymyxa*. The *Paenibacillus* sp. MVY-024 strain performed a positive effect on nitrogen changes in the soil and promoted spring wheat productivity parameters. After fermentation medium and fermentation parameters optimization, a cheaper medium composition was obtained, and the fermentation protocol was constructed. The most suitable sources for *Paenibacillus* sp. MVY-024 strain cultivation are sugarcane molasses and yeast extract, with the fermentation conditions of pH in the range of 6.0–7.0, temperature of 32 °C, and air flow of 0.4 vvm. This strain has a high potential as microbial fertilizer, because of the beneficial effect on plants and easy application in the industry. However, more investigation is needed to reveal the plant growth promotion mechanism in *Paenibacillus* sp. MVY-024 and to verify the safety of the release of this strain into the environment.

## Figures and Tables

**Table 1:** Nitrogen and carbon source optimization.

Carbon Source Optimization				Nitrogen Source Optimization			
Carbon Source, g L^−1^		Nitrogen Source, g L^−1^		Nitrogen Source, g L^−1^		Carbon Source, g L^−1^	
Glucose	40	Yeast extract	10	Soybean peptone	10	Molasses	57
Sucrose	40	Yeast extract	10	Carbamide	10	Molasses	57
Glycerol	40	Yeast extract	10	Ammonium suphate	10	Molasses	57
Mannitol	40	Yeast extract	10	Casein peptone	10	Molasses	57
Molasses	57	Yeast extract	10	Agropeptone	10	Molasses	57
Starch	40	Yeast extract	10	Yeast extract	10	Molasses	57
				Meat extract	10	Molasses	57
Molasses concentration optimization				Yeast extract concentration optimization			
Molasses	25	Yeast extract	10	Yeast extract	5	Molasses	57
Molasses	50	Yeast extract	10	Yeast extract	10	Molasses	57
Molasses	100	Yeast extract	10	Yeast extract	15	Molasses	57
Molasses	200	Yeast extract	10	Yeast extract	20	Molasses	57

**Table 2:** Growth of isolates in semi-selective media, no growth (−), weak growth (+), medium growth (++), and intensive growth (+++).

Isolates	Ashby’s	Winogradsky’s	NF	NFB	Gram Reaction	Endospore Formation
**Isolates from soil**						
Isolate S1	+++	+	+++	+	+	+
Isolate S2	++	++	+	+	−	−
Isolate S3	++	++	+++	+	−	−
Isolate S4	−	−	+	−	+	+
Isolate S5	++	+++	++	−	+	+
Isolate S6	+	++	+	−	+	+
Isolate S7	++	++	+++	+	+	+
Isolate S8	+	+	+	−	+	+
Isolate S9	+	++	++	−	+	+
**Isolates from pea root nodules**						
Isolate R1	+	+	++	−	+	+
Isolate R2	−	−	++	−	+	+
Isolate R3	+++	+	++	+	−	−
Isolate R4	+++	−	+++	+	−	−

**Table 3:** Isolates’ BLAST analysis results.

Isolate	Hit Taxon	Similarity, %	Sequence Length, bp	Accession
S1	*Paenibacillus peoriae* DSM 8320(T)	99.28	1390	AJ320494
S2	*Ensifer meliloti* LMG 6133(T)	100.00	1320	X67222
S3	*Ensifer medicae* WSM419(T)	99.70	1320	CP000738
S4	*Bacillus siamensis* KCTC 13613(T)	99.86	1390	AJVF01000043
S5	*Bacillus mojavensis* RO-H-1(T)	99.93	1390	JH600280
S6	*Bacillus paramycoides* NH24A2(T)	99.93	1400	MAOI01000012
S7	*Paenibacillus polymyxa* ATCC 842(T)	99.71	1380	AFOX01000032
S8	*Bacillus paralicheniformis* KJ-16(T)	100.00	1390	KY694465
S9	*Bacillus siamensis* KCTC 13613(T)	99.93	1390	AJVF01000043
R1	*Bacillus zanthoxyli* 1433(T)	99.51	1421	KX865140
R2	*Bacillus velezensis* CR-502(T)	99.85	1399	AY603658
R3	*Lelliottia amnigena* NBRC 105700(T)	99.42	1379	BCNN01000001
R4	*Rhizobium leguminosarum* USDA 2370(T)	100.00	1334	MRDL01000029

**Table 4:** The alteration of soil ammonium and total mineral N (mg kg^−1^) as affected by *Paenibacillus* spp. strains in different time lags after microorganism application. The values marked with the same letter have no significant differences at *p* ≤ 0.05.

No.	Treatment	Soil N-NH_4_, after 4 Days	Soil N-NH_4_, after 2 Weeks	Soil N-NH_4_, after 2 Months	Soil N_min_, after 2 Months
1.	Control	36.5 ab	36.5 a	1.12 a	3.13 ab
2.	*Paenibacillus* sp. S7	42.3 b	47.1 b	2.87 b	4.48 b
3.	*Paenibacillus* sp. S1	41.6 ab	48.1 c	0.29 a	1.48 a
Probability Pr>F		0.1415	0.001	0.026	0.042

**Table 5:** Spring wheat productivity and yield quality under the effect of *Paenibacillus* spp. strains. The values marked with the same letter have no significant differences at *p* ≤ 0.05.

No.	Treatment	N Yield in Grain, g^−1^ per Plant	Leaf Chlorophyll, SPAD	Grain Yield per Plant, g	TKW, g	Protein in Grain, %	Kernels per Spike, Unit
1.	Control	0.0355 ab	45.8 a	1.42 a	40.9 a	11.5 a	34.7 a
2.	*Paenibacillus* sp. S7	0.0361 b	46.8 b	1.54 b	43.1 b	12.8 b	36.0 b
3.	*Paenibacillus* sp. S1	0.0297 a	45.1 a	1.46 ab	40.4 a	11.1 a	36.2 b
Probability Pr > F		0.041	0.025	0.361	0.196	0.118	0.831

## Data Availability

The genome sequence of *Paenibacillus* sp. MVY-024 was deposited in the NCBI GenBank under the accession number MT900581.
